# Hyperglycemia and insulin resistance in COVID-19 versus non-COVID critical illness: Are they really different?

**DOI:** 10.1186/s13054-021-03861-6

**Published:** 2021-12-17

**Authors:** Lies Langouche, Greet Van den Berghe, Jan Gunst

**Affiliations:** Clinical Department and Laboratory of Intensive Care Medicine, Department of Cellular and Molecular Medicine, KU Leuven, Herestraat 49, 3000 Leuven, Belgium

**Keywords:** COVID-19, Hyperglycemia, Insulin, Diabetes

## Background

**Fig. 1:**
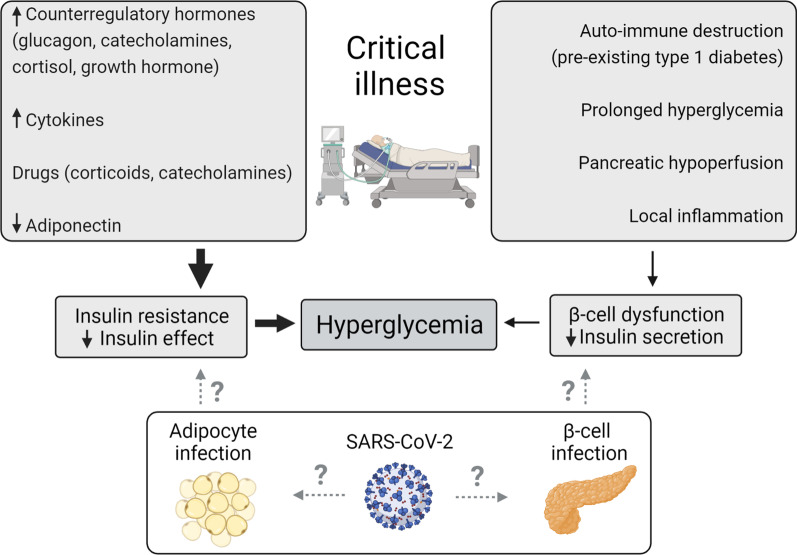
Potential pathophysiological mechanisms of hyperglycemia in COVID-19 and non-COVID-induced critical illness. The figure was created with BioRender.com

Hyperglycemia frequently develops in patients with severe COVID-19, regardless of preadmission diabetes status, as in non-COVID critically ill patients [1, 2]. In non-COVID patients, stress hyperglycemia has been attributed to insulin resistance due to elevated counterregulatory hormones, cytokines, and drugs including steroids, although beta-cell dysfunction through prolonged hyperglycemia, poor beta-cell reserve, hypoperfusion and inflammation may co-exist in some patients (Fig. 1) [3]. As in non-COVID patients, numerous observational studies have associated more severe hyperglycemia and increased glucose variability with poor outcome in COVID-19 patients [1, 2, 4, 5]. However, causality remains unclear, since insulin resistance and resultant hyperglycemia closely relate to illness severity [1, 6]. In this regard, a recent observational study also associated insulin treatment with increased mortality of COVID-19 [7]. Evidently, observational studies have an inherent risk of residual confounding, whereby the ideal glucose target can only be derived from adequately powered randomized controlled trials (RCTs).

Despite substantial similarities with non-COVID critical illness, some investigators have suggested that COVID-19 patients may be at increased risk of more severe hyperglycemia due to virus-mediated effects on beta-cell function and/or insulin sensitivity (Fig. 1) [8–11], which could predispose COVID-19 patients to diabetic emergencies. On the other hand, patients with severe COVID-19 more commonly have pre-existing diabetes and/or increased body-mass index as risk factors for insulin resistance, which is aggravated by routine steroid treatment [2, 12]. Clinical evidence confirming an increased incidence of diabetic emergencies related to COVID-19 surges is inconsistent, as are mechanistic studies on potential virus-induced alterations in glucose homeostasis [2, 8–11].

In a recent mechanistic study, Reiterer et al. reported that COVID-19-associated hyperglycemia may be driven through viral adipocyte infection, resulting in reduced release of adiponectin—a glucoregulatory hormone—﻿, and secondary insulin resistance [11]. Indeed, in animal and in vitro studies, adipocytes could be infected by SARS-CoV-2, which was associated with decreased adiponectin expression. Moreover, in a relatively small human study (*N* = 101), patients with COVID-19-associated acute respiratory distress syndrome had lower circulating adiponectin and elevated C-peptide over glucose—potentially indicating insulin resistance and beta-cell reserve—as compared to intensive care patients without COVID-19, who were classified as having (predominantly) beta-cell failure as cause of hyperglycemia [11]. Yet, it remains unclear whether these findings reveal a specific pathophysiological response to SARS-CoV-2, since low adiponectin concentrations have been reported in non-COVID critical illness as well [13]. Moreover, numerous potential confounders warrant caution. First, patients were not matched for baseline characteristics, with more diabetics in the non-COVID cohort and—presumably—a higher illness severity in the COVID-19 cohort, in view of the higher peak glucose concentrations observed in these patients. Hence, baseline imbalance could explain the claimed differences in insulin resistance versus beta-cell failure between cohorts. Second, circulating glucose, C-peptide and adiponectin are affected by nutrition and insulin treatment [14, 15], which was not reported, respectively differed between the groups, and time of blood sampling was not standardized. Finally, the used definitions of insulin resistance and beta-cell failure are contentious. Insulin resistance was defined as the absence of beta-cell failure, although both conditions may co-exist. Moreover, the cutoff of C-peptide over glucose was not validated, and in addition to effects of insulin treatment on C-peptide and glucose, concentrations were not measured concomitantly. Finally, the finding of predominant beta-cell failure in non-COVID critical illness contradicts with preceding evidence [3, 14], which demonstrated ubiquitous insulin resistance and which questions the used tool to define insulin resistance in this study.

In contrast, other mechanistic studies have put forward virus-induced beta-cell dysfunction or damage as potential mediator of hyperglycemia in COVID-19 [8–10]. In human islets, SARS-CoV-2 was able to infect beta-cells, leading to cell death [9]. Also autopsy studies in patients dying with COVID-19 revealed presence of SARS-CoV-2 transcript and/or antigen in beta-cells of some, but not all patients [8–10]. However, apart from the low number of included patients, several issues warrant caution not to overinterpret these findings, including the suboptimal tissue quality due to autolysis, conflicting data regarding the pancreatic expression of SARS-CoV-2 receptors and the beta-cell selectivity of viral damage, and the lack of in vivo evidence [8–10]. If virus-mediated beta-cell death would be a primary mechanism of hyperglycemia in severe COVID-19, one would expect persistent insulin need in the majority of patients, which is not the case [2]. Whether COVID-19 associates with an increased incidence of persistent diabetes mellitus or not, is currently being investigated [2]. Interestingly, a recent mechanistic study found potentially reversible beta-cell transdifferentiation rather than cell death in human islets exposed to SARS-CoV-2, characterized by lower expression of insulin and upregulated expression of alpha-cell markers [10]. Also in this case, it remains unclear whether such transdifferentiation also occurs in vivo.

From a clinical perspective, the question is to what extent COVID-19-associated hyperglycemia should be treated, for which there is yet no solid RCT evidence. Also in non-COVID critical illness, the ideal glucose target remains debated [1]. Our research group showed via three RCTs improved morbidity and mortality of critically ill children and adults by maintaining blood glucose with insulin in the healthy, normal range. Clinical benefit was subsequently attributed to prevention of glucose overload and associated mitochondrial damage. In contrast, the largest multicenter RCT in critically ill adults found an opposite impact of tight glucose control on mortality, which was attributed to an increased incidence of hypoglycemia. The differences between the subsequent RCTs could be explained by differences in accuracies of the glucose control protocols and differences in feeding strategies [1]. The multicenter TGC-fast RCT is currently investigating whether tight glucose control performed with a validated protocol that minimizes the incidence of hypoglycemia is still beneficial in the absence of early full feeding (clinicaltrials.gov NCT03665207).

## Conclusions

Stress hyperglycemia and insulin resistance are characteristic of acute critical illness. It remains unclear if COVID-19-associated hyperglycemia and insulin resistance is more severe than in non-COVID patients with similar disease severity, and (in that case), whether this is mediated by viral infection of beta-cells and/or adipocytes. As in non-COVID critically ill patients, the ideal blood glucose target remains to be defined.

## Data Availability

Not applicable.

## Abbreviation

RCT: Randomized controlled trial
